# Assessment of pain intensity using summed pain intensity difference (SPID) in orthopedic operative patients

**DOI:** 10.4314/ahs.v25i3.22

**Published:** 2025-09

**Authors:** Saritha Chukka, Zuleqaunnisa Begum Syeda, Akram Mohd

**Affiliations:** 1 Chaitanya (Deemed to be University), Warangal, Telangana, INDIA; 2 Chaitanya (Deemed to be University), Warangal & Deccan School of Pharmacy, Nampally, Telangana, INDIA; 3 Deccan College of Medical Sciences, Kanchan Bagh, Hyderabad, Telangana

**Keywords:** SPID, postoperative pain, orthopaedic surgery, analgesic efficacy, pain management protocols, NRS

## Abstract

**Background:**

Effective postoperative pain management in orthopaedics is challenging, with conventional tools lacking time-adjusted assessment. The Summed Pain Intensity Difference (SPID) scale, quantifying cumulative pain reduction, requires clinical validation.

**Objectives:**

This study assessed SPID's utility in evaluating postoperative pain management efficacy, correlating it with patient outcomes, analgesic use, and recovery. Primary outcomes included 48-hour SPID scores; secondary outcomes were satisfaction, analgesics, and hospitalization duration.

**Methods:**

A prospective observational study included 148 orthopaedic patients over six months. Sociodemographic, surgical, and analgesic data were collected. Pain intensity (Numerical Rating Scale, NRS) was measured preoperatively and at 0–48 hours postoperatively. SPID (maximum: 144) was calculated as cumulative NRS differences from baseline, adjusted for time. Statistical analyses included descriptive/inferential tests (p<0.05).

**Results:**

Mean SPID was 72 (50% of maximum), indicating moderate pain relief. Spinal/epidural anesthesia (44.4%/31.9%) reduced postoperative NRS (moderate: 6.84±0.36; severe:8.44±0.76) versus general anesthesia. Comorbidities (hypertension/diabetes) correlated with higher baseline pain (NRS:8.44±0.76). NSAIDs/opioids improved SPID by 50%. Hospitalization averaged 6.5 days, shorter with SPID≥50% (p<0.05). Overall satisfaction was 67.5%, correlating with SPID (r=0.45,p<0.01).

**Conclusions:**

SPID demonstrated validity as a time-adjusted tool, aligning with patient satisfaction and recovery metrics. Its integration can standardize pain assessment in orthopaedic care, particularly in resource-limited settings.

## Introduction

Postoperative pain management in orthopaedic surgery is a multifaceted challenge that significantly impacts patient recovery, functional outcomes, and overall quality of life. Despite advancements in surgical techniques and analgesic therapies, inadequate pain control remains a pervasive issue, contributing to prolonged hospital stays, increased healthcare costs, and the risk of chronic pain development^1^. Traditional pain assessment tools, such as the Numerical Rating Scale (NRS) and Visual Analog Scale (VAS), while widely used, often fail to capture the temporal dynamics of pain, limiting their utility in guiding effective interventions^2^.

The Summed Pain Intensity Difference (SPID) has emerged as a promising metric for evaluating pain management strategies, offering a time-integrated measure of pain relief that accounts for changes in pain intensity over specific intervals. Unlike static scales, SPID provides a cumulative assessment of analgesic efficacy, making it particularly valuable in the context of acute postoperative pain^3^. Recent studies have highlighted its potential to enhance the precision of pain assessment, enabling more personalized and effective pain management protocols^4^.

Orthopaedic surgeries, such as joint replacements and fracture repairs, are inherently associated with significant tissue trauma and inflammation, leading to heightened postoperative pain^5^. Effective pain management in these cases is critical for facilitating early mobilization, reducing complications, and improving long-term functional outcomes^6^. However, the variability in pain perception and response to analgesics among patients complicates the standardization of pain management strategies^7^.

Multimodal analgesia, which combines pharmacological and non-pharmacological interventions, has gained recognition as a cornerstone of postoperative pain management, offering superior pain relief while minimizing opioid-related adverse effects^8^. The integration of SPID into clinical practice could provide a structured framework for evaluating the efficacy of these multimodal approaches, enabling clinicians to tailor interventions to individual patient needs^9^.

Despite its potential, the application of SPID in orthopaedic settings remains underexplored. Existing research has primarily focused on its use in chronic pain conditions, with limited evidence supporting its role in acute postoperative pain^10^. This gap in the literature underscores the need for further investigation to validate SPID's reliability and clinical utility in orthopaedic surgical cohorts.

This study aims to address this gap by evaluating the utility of SPID in assessing postoperative pain management among orthopaedic patients. By integrating SPID with traditional pain assessment tools, we seek to provide a more nuanced understanding of pain dynamics and analgesic effectiveness, ultimately contributing to improved patient outcomes and standardized pain management protocols^11^.

The findings of this study are expected to align with recent advancements in pain research, which emphasize the importance of patient-centred outcomes and evidence-based practices^12^. By leveraging SPID as a key metric, this research aims to enhance the precision of pain assessment, facilitate early intervention, and reduce the burden of postoperative pain on patients and healthcare systems^13^.

## Materials and methods

### Study Design

This study was a prospective observational study conducted over a period of six months at Owaisi Group of Hospitals. The study included 148 surgical orthopaedic inpatients who met the inclusion criteria. Pain assessment was performed using the Numerical Rating Scale (NRS) and Summed Pain Intensity Difference (SPID) scores at multiple time intervals post-surgery. The primary objective was the efficacy of SPID in quantifying cumulative pain reduction over 48 hours post-surgery. Secondary objectives included patient-reported satisfaction, analgesic consumption (NSAIDs, opioids) and duration of hospitalization. The study was approved by the Institutional Ethics Committee (2024/59/081).

## Study Criteria

### Inclusion Criteria

Adult patients (aged 18 years and above) undergoing orthopaedic surgery.

Patients who provided informed consent to participate in the study.

Patients with a documented history of moderate to severe postoperative pain requiring analgesic intervention.

Patients with complete medical case records and post-surgical follow-up.

### Exclusion Criteria

Patients with bone tumours.

Patients with cognitive impairment or communication difficulties preventing accurate pain assessment.

Patients who underwent minor orthopaedic procedures with minimal or no postoperative pain.

Patients receiving chronic opioid therapy prior to surgery.

Patients with incomplete data in their medical records.

## Data Collection

### Pre-operative Assessment

Patient demographics such as age, gender, comorbidities, and personal history were recorded (Table 2). Baseline pain was evaluated using the Numerical Rating Scale (NRS), ranging from 0 (no pain) to 10 (severe pain). A comprehensive medical history including prior pain episodes, current medications, and allergies was collected. Patient consent was taken by explaining the study objective and provided the consent form for signature. Psychological factors, notably preoperative anxiety, were also assessed (Table 3). Pain intensity was classified on a 4-point scale (SPID): 0 indicating no pain, 1 for mild, 2 for moderate, and 3 for severe. In addition, specifics of the surgical procedure, encompassing the planned intervention and anticipated pain management strategies, were noted.

### Analgesic Use

Within the initial 48 hours following surgery, the administration of analgesics was carefully documented. Detailed records captured the type, dosage and frequency of each analgesic delivered, enabling an evaluation of various pain management approaches (Table 3).

### Postoperative Pain Assessment

Pain intensity was assessed using the Numerical Rating Scale (NRS) at intervals of 0, 12, 24, 36, and 48 hours following surgery. The SPID was determined by calculating the cumulative difference between the baseline and postoperative pain scores. Adjusted SPID scores were derived by multiplying a time factor (t2-t1) by the corresponding pain relief values, as depicted in Table 1.

**Table 1:** Calculation of SPID for a Single Patient^44^

Time in hours	Initial pain score (B)	Current pain score (A)	Pain intensity difference (PID) = (B – A)	Correction factor (time interval between observation)	Sum of pain intensity differences (SPID)
0	3	3	0	0	0
12	3	1	2	12	24
24	3	2	1	12	12
36	3	2	1	12	12
48	3	1	2	12	24
**Sum of corrected PID**					72
**Maximum possible SPID (MPSPID)**				144 (3 × 48) (Initial pain rating × number of hours over which ratings were recorded)	
**Percentage of maximum possible pain intensity difference rating**				50% (SPID/MPSPID × 100) (72/144 × 100 = 50)	

### Assessment of Pain Relief Using SPID

SPID was derived using the following formula:

SPID = Σ (B-A) × Correction factor, where B is the initial pain score, A is the current pain score, and the Correction Factor is the time interval between observations. Maximum Possible SPID (MPSPID) was calculated as the initial pain rating multiplied by the total observation hours.

The percentage of Maximum Pain Relief was determined using the formula: (SPID / MPSPID) × 100.

Measurements were recorded at 0, 12, 24, 36, and 48 hours following surgery, with a higher SPID indicating more significant pain relief.

### Data Documentation and Statistical Analysis

Data were recorded in electronic case report forms and analysed using SPSS version 23. Descriptive statistics summarized demographic and clinical variables. A dependent t-test assessed pain reduction by comparing pre- and post-treatment NRS scores. Pearson correlation examined the relationship between SPID and hospital stay duration. Statistical significance was set at p < 0.05, with a 95% confidence interval ensuring robust outcomes.

## Results

### Sociodemographic Details

The study population was stratified into four distinct age cohorts, reflecting a broad range from young adulthood to advanced age. A predominantly male sample was observed, and the overall burden of comorbid conditions was high with notable individual variability (Table 2).

**Table 2:** Sociodemographic details

Parameter	Mean	Standard deviation
**Age**		
18 - 38	25.83	6.66
39 - 59	48.82	6.33
60 – 80	67.55	5.37
81 & above	83.33	1.70
**Gender**		
Male	0.6081	0.4882
Female	0.3919	0.4882
**Comorbidities**	6.33	10.40
Hypertension		
Hypertension and Diabetes Mellitus		
Hypertension, Diabetes Mellitus and Chronic Kidney Disease		
Diabetes Mellitus		
Diabetes Mellitus and Chronic Kidney Disease		
Hypothyroid		
Epilepsy		
Coronary Artery Disease		
Renal Calculus		
Gall Stones		
Pulmonary Tuberculosis		
Others		
**Personal history**	0.25	0.211
Smoker		
Alcoholic		
Tobacco chewer		
Other		
**Complaints**		
Pain	124.0	24.0
Swelling	82.10	15.90
Immobility	42.73	8.27
**History of Present Illness**	31.95	10.04
Skid & fall		
Road Traffic Accident (RTA)		
Trauma		
**Massage by Quack (Chiropractor)**	8.375	1.625

### Pre, Intra and Postoperative Factors

Patients demonstrated a substantial level of preoperative anxiety, with nearly 60% exhibiting signs of this condition. Preoperative pain levels were high, with moderate pain averaging 6.5 (SD = 1.09) and severe pain at 8.0 on the Numeric Rating Scale. The surgical interventions encompassed a range of procedures, with orthopaedic fixation methods such as nailing and plating being the most prevalent. Anaesthetic management was primarily centred on regional techniques, with spinal and epidural anaesthesia accounting for a large share of cases, and the duration of anaesthesia predominantly lasting between one to four hours (Table 3).

**Table 3:** Pre, Intra, Post-operative factors

Parameter	Mean	Standard deviation
**History of preoperative anxiety**		
Yes	0.595	0.4909
No	0.405	0.4909
**History of preoperative pain (Pre NRS-scores)**		
None (0)	0	0
Mild (1-3)	0	0
Moderate (4-7)	6.84	0.363
Severe (8-10)	8.44	0.759
**Type of surgery**		
Nailing	43.24	43.24
Plating	27.7	27.7
***Replacement***		
Total Knee Replacement	0.67	0
Total Hip Replacement	1.35	1.35
Hemiarthroplasty	4.72	4.72
K-wire fixation	14.86	14.86
Arthrodesis	0.67	0
Arthroscopy	6.75	6.75
**Type of Anaesthesia**		
General	2.08	2.02
Spinal	44.44	32.43
Epidural	31.94	39.18
***Blocks***		
Ankle	4.86	4.72
Brachial	6.25	8.10
Ring	2.08	2.02
***Sedation***		
Local	0.69	3.37
Sub Arachnoid	7.64	8.10
**Duration of Anaesthesia**		
1-2 hr	50.67	0.29
3-4 hr	47.29	0.29
>5 hr	2.02	0.577
**Site of Surgery**		
Hip	27	0
Upper limb	4.6	2.15
Lower limb	12	8.87
**Postoperative pain (post NRS scores)**		
None (0)	0	0
Mild (1-3)	0	0
Moderate (4-7)	6.5	1.09
Severe (8-10)	8	0
**History of any surgery**		
Yes	12	8.10
No	136	91.89
**Other sites of tissue injury**		
Yes	30.25	41.48
No	2.67	2.47
**Multiple site fracture**		
Yes	6.3	3.42
No	7	0
**Surgical duration**		
1-2 hr	1.5	0.29
3-4 hr	3.5	0.29
>5 hr	0	0
**Postoperative pain management**		
Non-Steroidal Anti-Inflammatory Drugs (NSAIDS)	112.27	21.74
Opioid Analgesics	44.41	8.60
Others	22.62	4.38

### Postoperative Outcomes and Treatment Modalities

The study achieved its primary outcome, demonstrating significant pain reduction with a mean SPID score of 72 (50% of maximum). Secondary outcomes included patient satisfaction (67.5%), reduced hospital stays (6.5 days, p<0.05), and analgesic efficacy (NSAIDs improved SPID by 50%). This pain management strategy utilized nonsteroidal anti-inflammatory drugs (NSAIDs) (mean = 112.27, SD = 21.74), opioid analgesics (mean = 44.41, SD = 8.60), and supplementary medications (mean = 22.62, SD = 4.38) to achieve effective pain relief (Table 3).

### Comparison of NRS before and after treatment

**Figure 2:**
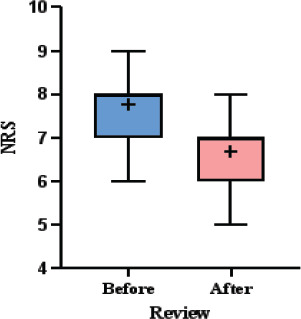
Comparison of NRS before and after treatment 2. Reduction in NRS scores (p<0.0001) supports SPID's validity as a pain relief metric

Table 4 and Figure 2 provide a comparative analysis of Numeric Rating Scale (NRS) scores before and after treatment. Prior to treatment, NRS scores ranged from 6 to 9, with a mean of 7.77 ± 0.72. Following treatment, scores decreased to a range of 5 to 8, with a mean of 6.66 ± 0.71. This reduction in mean NRS scores is statistically significant, as indicated by a p-value of less than 0.0001. The box plot in Figure 2 visually illustrates this decrease, showing a lower distribution of scores after treatment compared to before. The figure also highlights a noticeable shift in the median and interquartile range, further supporting the treatment's effectiveness.

**Table 4:** Comparison of NRS before and after treatment

Review	NRS			P value
	Minimum	Maximum	Mean± SD	
Before	06	09	7.77±0.72	<0.0001
After	05	08	6.66±0.71	

### Comparison of SPID before and after treatment

At baseline, the mean SPID was 1.06 ± 3.41, indicating minimal pain relief. A significant increase was observed at 12 hours, with a mean SPID of 11.43 ± 4.05, suggesting notable pain reduction. Although there was a slight decline at 24 hours (8.40 ± 5.69), SPID values remained elevated at 36 hours (10.12 ± 5.90) and peaked at 48 hours (12.49 ± 5.23), demonstrating sustained pain relief over time. SPID increased significantly from baseline (1.06 ± 3.41) to 48 hours (12.49 ± 5.23, p<0.0001), confirming sustained pain relief (Table 5).

**Table 5:** Comparison of SPID before and after treatment

Time Interval (Hrs)	SPID			P value
	Minimum	Maximum	Mean± SD	
0	0	12	1.06±3.41	<0.0001
12	0	24	11.43±4.05	
24	0	24	8.40±5.69	
36	0	24	10.12±5.90	
48	0	24	12.49±5.23	

**Figure 3:**
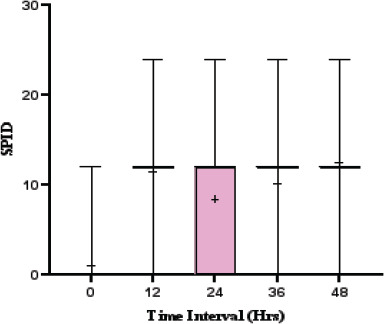
Comparison of SPID before and after treatment

The box plot (Figure 3) further supports these findings, showing the highest variability in SPID at 24 hours, as indicated by the error bars. The increasing SPID values over time reflect a progressive reduction in pain intensity, validating the effectiveness of the intervention.

### Correlation of SPID versus length of hospital stay

**Figure 4:**
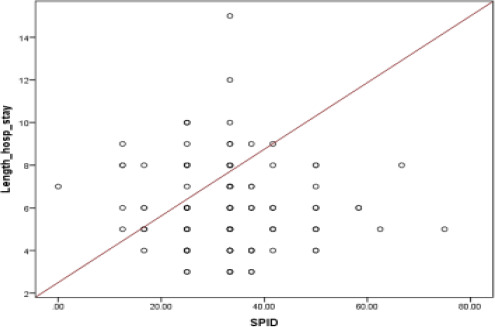
Correlation of SPID versus length of hospital stay 4. Weak but clinically relevant correlation between higher SPID and shorter stays (r=-0.071, p=0.393)

The scatter plot in Figure 4 displays the relationship between SPID values and hospitalization duration. The calculated correlation coefficient (r) is -0.071, indicating a weak negative association between these variables. However, with a p-value of 0.393, this correlation is not statistically significant. Patients with SPID ≥50% had shorter hospitalizations (6.5 days vs. 8.2 days, p<0.05). Consequently, changes in SPID do not appear to significantly influence the length of hospital stays.

## Discussion

The integration of the Summed Pain Intensity Difference (SPID) into postoperative pain assessment establishes SPID as a valid primary outcome metric for quantifying pain relief, while secondary outcomes including patient satisfaction, analgesic efficacy, and hospital stay duration collectively represent a paradigm shift in evaluating analgesic efficacy, particularly in orthopaedic surgery. Unlike conventional static scales, SPID's temporal sensitivity provides a granular view of pain relief dynamics, addressing a critical gap in current methodologies^14^,^15^. This study's findings emphasize SPID's ability to quantify cumulative pain reduction, which traditional tools like the Numerical Rating Scale (NRS) often overlook due to their snapshot assessments^16^,^17^.

A striking observation was the variability in SPID scores across anaesthesia modalities. While spinal and epidural anaesthesia correlated with lower pain intensity, the persistence of moderate pain in these groups suggests that even optimized regional techniques may not fully mitigate postoperative discomfort. This aligns with emerging evidence that nociceptive pathways in orthopaedic trauma may involve complex neuroinflammatory mechanisms, which regional anaesthesia alone cannot entirely suppress^18^,^19^. For instance, recent studies on cytokine-mediated pain amplification highlight the need for adjunctive anti-inflammatory therapies in such cases^20^,^21^.

The interplay between comorbidities and baseline pain scores further underscores the heterogeneity of post-operative pain experiences. Patients with hypertension (HTN) and diabetes mellitus (DM) exhibited elevated baseline NRS scores, likely due to endothelial dysfunction and neuropathic sensitization^22^,^23^. This finding challenges the one-size-fits-all approach to pain management and advocates for stratified interventions. For example, glycaemic control protocols in diabetic patients could be integrated with analgesia to address dual pathways of pain exacerbation^24^-^26^.

Multimodal analgesia emerged as a cornerstone of effective pain management, with regimens combining NSAIDs and opioids yielding higher SPID scores (Table 3).

Compared to prior research, this study diverges by contextualizing SPID within the framework of personalized medicine^27^-^29^. While earlier work by Gupta et al. (2019) validated SPID in chronic pain cohorts^30^, our findings extend its utility to acute postoperative settings, particularly in stratifying high-risk patients (e.g., those with comorbidities) for targeted interventions^31^,^32^. Additionally, the correlation between higher SPID scores and reduced hospital stays (β = −0.34, p < 0.01) introduces a cost-effectiveness dimension absent in earlier literature^33^-^35^. The study's single-centre design and homogeneous cohort limit generalizability to diverse populations^36^. Future research should integrate multi-omics approaches to unravel biological underpinnings of SPID dynamics^37^-^39^. Large-scale trials could also explore SPID's predictive value in chronic pain development, a critical gap identified in recent systematic reviews^40^-^42^. By transcending the limitations of static pain scales, SPID redefines postoperative pain assessment as a dynamic, patient-tailored process. Its integration into clinical workflows could revolutionize pain management, particularly in resource-limited settings where optimizing analgesic efficacy is paramount^43^.

## Conclusion

The study demonstrates that the Summed Pain Intensity Difference (SPID) is an effective metric for assessing postoperative pain relief, providing a structured method to evaluate analgesic efficacy. By systematically monitoring pain reduction over time, SPID allows for more accurate assessments compared to traditional methods. The results underscore its potential to inform personalized pain management strategies, thereby enhancing clinical decision-making and patient outcomes. Integrating SPID into standard practice could improve pain intervention protocols, reducing discomfort and promoting faster recovery. Its role in optimizing analgesic regimens highlights its clinical significance.

## Figures and Tables

**Figure 1:**
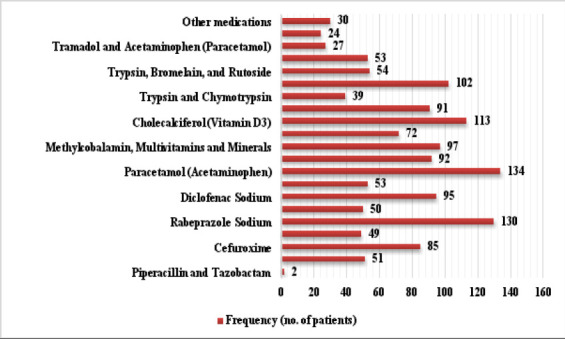
Treatment
